# Genetic Evaluation for Functional Longevity in Nellore Cattle

**DOI:** 10.1111/jbg.70067

**Published:** 2026-08-10

**Authors:** Gabriele de Souza Silva Fernandes, Dalila Ribeiro dos Santos, Arione Augusti Boligon, Mário Luiz Santana, Gilberto Romeiro de Oliveira Menezes, Denise Rocha Ayres, Annaiza Braga Bignardi, Rodrigo Junqueira Pereira

**Affiliations:** ^1^ Grupo de Melhoramento Animal de Mato Grosso (GMAT), Universidade Federal de Rondonópolis Rondonópolis MT Brazil; ^2^ Departamento de Zootecnia Universidade Federal de Pelotas (UFPEL) Pelotas RS Brazil; ^3^ Embrapa Gado de Corte Campo Grande MS Brazil

**Keywords:** early selection, random regression, reproduction, survival

## Abstract

Stayability (STAY) presents limitations in capturing genetic variability among animals with varying calving records up to 76 months of age. Furthermore, because STAY is measured late in life (typically at 6 years of age), it inherently prolongs generation intervals. To address this limitation, we evaluated an alternative approach using random regression models, which incorporate multiple phenotypes recorded throughout an animal's lifespan to generate predictive curves for breeding values. Using calving records from 179,001 Nellore cows, we compared two phenotypic expressions of longevity and aimed to identify the optimal random regression model for predicting breeding values. Ten specific ages were defined for functional longevity expressed as stayability (FLSTAY). At each age, a binary phenotype (1 or 0) was assigned based on whether the cow remained in the herd. Alternatively, for functional longevity associated with the number of calvings (FLNC), the cumulative number of calvings recorded up to each specific age was calculated. Models fitting Legendre orthogonal polynomials ranging from linear to cubic degrees were compared. The cubic polynomial proved most suitable for modelling FLSTAY, whereas the quadratic polynomial provided the best fit for FLNC. Predictive ability metrics of estimated breeding values for FLNC were superior to those for FLSTAY. Heritability estimates for FLNC (0.025–0.074) were consistently higher than those for FLSTAY (0.004–0.045). Similarly, genetic correlations across ages were generally stronger for FLNC, reaching 0.88 between 39 and 75 months (the traditional age for selection). These findings suggest the feasibility of applying random regression models to FLNC, allowing its use as an early selection strategy to improve functional longevity.

## 1 Introduction

Stayability (STAY) is widely incorporated into animal breeding programmes, because it predicts the genetic merit for cow longevity within the herd. The retention of cows in the herd is primarily associated with the total number of calves she produces over her lifetime. Consequently, STAY is the trait with the highest relative economic importance in selection indices, exerting a greater economic impact than productive traits such as weaning and carcass weights (Costa et al. 2017).

Despite some differences among breeding programmes, STAY is traditionally defined as the ability of cows to remain in the herd up to 76 months of age, provided they have calved at least three times (Silva et al. 2018). By 76 months of age, a cow has the potential to calve up to five times. However, the traditional STAY definition fails to phenotypically differentiate between cows with three, four, or five calvings, uniformly categorizing them as successes. Similarly, it does not distinguish between cows with one or two calvings, grouping them as failures. Furthermore, because STAY is measured late in an animal's life, it significantly delays the inclusion of progeny records in the genetic evaluation of sires (Jamrozik et al. 2013).

To address these limitations, several alternative approaches to evaluate stayability have been proposed. For instance, Snelling et al. (1995) analysed four STAY scenarios: the probability of a cow calving two, five, eight and eleven times after her first calving. Martinez et al. (2004) defined the trait as the length of productive life, measured by the number of days a cow survived from 1 to 6 years following her first calving. van Melis et al. (2007) investigated three definitions for STAY, assessing whether a cow calved annually up to 5, 6, or 7 years of age, given that her first calving occurred by 2 years of age.

Furthermore, Neves et al. (2012) proposed an alternative measure to traditional STAY: the accumulated number of calvings up to 53 months of age, ranging from 0 to 2. Brzáková et al. (2019) defined longevity as the number of calves produced up to four ages (e.g., 78, 90, 150 and 160 months), analysing them in single‐trait or multiple‐trait models. More recently, Oliveira et al. (2020) applied random regression models to evaluate functional longevity (FL) phenotypes spanning from 2 to 15 years of age. This approach aimed to enhance the differentiation of animal phenotypes and facilitate the early and accurate prediction of breeding values.

In a similar context, Aliloo et al. (2025) applied random regression models to compare two definitions of FL and assess the impact of using censored data on the prediction of sire breeding values in Australian Angus cattle. They demonstrated that excluding censored data from the analysis (i.e., removing daughters with incomplete productive lives) resulted in underestimated breeding values for the sires.

Passafaro et al. (2024) evaluated productive longevity by fitting a random regression model to longitudinal binary records of calving success or failure. The trait was defined based on the occurrence of regular and consecutive calvings from the second to the eighth parity up to 9 years of age, assuming that the first calving occurred at approximately 2 years of age.

Although a few studies have investigated longevity in Zebu breeds (Silva et al. 2018; Morales et al. 2024), none have applied a methodology that integrates cow longevity with reproductive performance. Therefore, the present study aimed to compare different definitions of FL in Nellore cows and to determine the most appropriate random regression model for fitting this trait. Finally, using the selected model, we estimated genetic parameters and performed the genetic evaluation of the animals.

## 2 Material and Methods

The dataset utilized in this study was provided by the Embrapa Beef Cattle Breeding Programme (Geneplus) and comprised records from Nellore cattle born across Brazil between 1950 and 2023. The original database contained 2,607,400 calving records from 814,630 cows.

During data quality control, females with any record of in vitro fertilization or embryo transfer were excluded from the analysis. The final dataset retained only cows with an age at first calving between 20 and 51 months, and with subsequent calving intervals greater than 305 days.

Two approaches were defined to assess cow longevity phenotypes. The first approach treated longevity as a binary trait, adapting the methodology proposed by Oliveira et al. (2020), whereas the second evaluated it as a cumulative trait.

To evaluate FL, 10 standard ages were established, ranging from 27 to 135 months, with 12‐month intervals. Phenotypes were generated based on the occurrence of calvings up to each of these predetermined ages, resulting in a vector containing up to 10 observations per cow. For FL expressed as stayability (FLSTAY), a binary phenotype of 1 was assigned if the cow had a recorded calving by age, and 0 if no calving was recorded. Conversely, for FL associated with the number of calvings (FLNC), the phenotype was defined as the cumulative number of calvings recorded up to each standard age.

For “young cows” (those that had not yet reached the older age classes by the end of the data collection period, and thus lacked the opportunity to express the complete phenotypes), the phenotype vector was truncated to match the number of possible calvings based on their maximum age at the time of data extraction. Cows that failed to produce their first calf by 51 months of age were excluded from the analysis. Furthermore, for cows that failed to calve for two consecutive years, any subsequent calving records were discarded. This restriction reflects standard management practices in commercial Brazilian Nellore herds, wherein females that fail to conceive for two consecutive mating seasons are routinely culled. The potential phenotypic vectors for each longevity definition, considering these constraints, are illustrated in Table 1.

**TABLE 1:** Phenotypic vectors under different circumstances according to the trait definition: Functional longevity expressed as stayability (FLSTAY) and functional longevity associated with the number of calvings (FLNC).

Cow	FLSTAY	FLNC	Profile description
A	[1,1,1,1,1,1,1,1,1,1]	[1,2,3,4,5,6,7,8,9,10]	Cow with calving records at all ages.
B	[1,1,0,1,1,1,0,1,1,1]	[1,2,2,3,4,5,5,6,7,8]	Cow with missed calvings at the 3rd and 7th ages.
C	[1,1,1,1,0,0,0,0,0,0]	[1,2,3,4,4,4,4,4,4,4]	Cow with calvings only up to the 4th age, or more than 2 consecutive failures, failing at the 5th and 6th ages.
D	[1,1,1,1]	[1,2,3,4]	Young cow whose maximum age corresponds to the 4th age, possessing all calving records up to that point.

Contemporary groups were defined by combining the farm, year, and season of the cow's birth, along with the farm of her first calving. To ensure an unbiased comparison between the two FL definitions, the data quality control protocol was dictated by the more restrictive filtering requirements of the binary trait (FLSTAY). Groups containing fewer than ten cows, lacking phenotypic variability, or consisting solely of daughters from a single sire were excluded from the analysis. Therefore, the same subset of animals and operational records were utilized to evaluate both FLSTAY and FLNC, eliminating any potential confounding effects or selection bias arising from asymmetric data structures between the two traits. Following data quality control, descriptive statistics were calculated for each trait definition (Table 2).

**TABLE 2:** Number of animals with phenotype (n), mean, standard deviation (SD), minimum (Min), and maximum (Max) values for functional longevity expressed as stayability (FLSTAY) and functional longevity associated with the number of calvings (FLNC) according to predetermined ages.

Age (months)	*N*	FLSTAY				FLNC			
		Mean	SD	Min	Max	Mean	SD	Min	Max
27	130,086	0.11	0.32	0	1	0.11	0.32	0	1
39	129,041	0.72	0.45	0	1	0.82	0.49	0	2
51	124,342	0.57	0.50	0	1	1.38	0.53	1	3
63	117,586	0.43	0.49	0	1	1.80	0.77	1	4
75	111,507	0.34	0.47	0	1	2.13	1.06	1	5
87	105,171	0.26	0.44	0	1	2.39	1.33	1	6
99	98,019	0.20	0.40	0	1	2.60	1.58	1	7
111	91,094	0.15	0.35	0	1	2.76	1.81	1	8
123	84,072	0.11	0.31	0	1	2.88	2.00	1	9
135	76,629	0.08	0.26	0	1	2.96	2.16	1	10

To evaluate FLSTAY and FLNC, the following random regression model was applied:

$$y_{\text{ijklt}}={\mathrm{YS}}_{\mathrm{it}}+\sum_{m=0}^{o-1}\beta_{\mathrm{km}}\varphi_{\mathrm{mt}}+\sum_{m=0}^{o-1}\alpha_{\mathrm{lm}}\varphi_{\mathrm{mt}}+\sum_{m=0}^{o-1}\delta_{\mathrm{lm}}\varphi_{\mathrm{mt}}+\varepsilon_{\text{ijklt}},$$

where $y_{\text{ijklt}}$ represents the FLSTAY or FLNC phenotype of cow *l* at age *t* (*t* = 27, …, 135 months); ${\mathrm{YS}}_{\mathrm{it}}$ is the systematic effect of the *i*‐th birth year‐season subclass at age *t*; $\beta_{\mathrm{km}}$ is the *m*‐th regression coefficient for the random effect of contemporary group *k*; $\alpha_{\mathrm{lm}}$ and $\delta_{\mathrm{lm}}$ are the *m*‐th regression coefficients for the random additive genetic and permanent environmental effects of cow *l*, respectively; $\varphi_{\mathrm{mt}}$ is the *m*‐th Legendre polynomial evaluated at age *t*; and $\varepsilon_{\text{ijklt}}$ is the random residual effect associated with each phenotypic observation. The random regression models were fitted using orthogonal Legendre polynomials of degree 1 to 3 (*o* = 2 to 4 coefficients, respectively). In each model, the same degree was used for contemporary group, additive genetic, and permanent environmental effects, so that each effect had the same opportunity to explore the spaces of the curves (Jamrozik et al. 2013). A heterogeneous residual variance structure was adopted, assigning a specific residual variance to each age.

In matrix notation, the model can be written as follows:

y=Xb+Cg+Za+Wp+e,

where **y** is the vector of observations; **b** is the vector of systematic effect *YS*; **g**, **a**, **p** and **e** are the vectors of random contemporary group, additive genetic, permanent environmental, and residual effects, respectively; **X**, **C**, **Z** and **W** are incidence matrices corresponding to the observations for systematic, contemporary group, additive genetic and permanent environmental effects, respectively. The following assumptions were defined for this model:

$$y|b,g,a,p,R\sim \mathrm{NMV}\left(\mathrm{Xb}+\mathrm{Cg}+\mathrm{Za}+\mathrm{Wp},R\right),\mathrm{Var}\left[\begin{array}{c} g\\ a\\ p\\ e \end{array}\right]=\left[\begin{array}{cccc} \Lambda_{G}⨂I & 0 & 0 & 0\\ 0 & \Lambda_{A}⨂A & 0 & 0\\ 0 & 0 & \Lambda_{P}⨂I & 0\\ 0 & 0 & 0 & R \end{array}\right],$$

where $\Lambda_{G}$, $\Lambda_{A}$ and $\Lambda_{P}$ are contemporary group, additive genetic and permanent environmental covariance matrices between the regression coefficients, respectively; **R** is the 10 × 10 diagonal residual variance matrix, with one specific variance for each age; **A** is the numerator relationship matrix between animals; **I** is an identity matrix, and ⊗ is the Kronecker product operator between matrices.

The analyses were performed via Bayesian inference using the GIBBSF90+ program (Lourenco et al. 2022). The following prior distributions were assumed for the model:

b∝constant,

$$g\;|\;\Lambda_{G}\sim \mathrm{MVN}\left(0,\Lambda_{G}⊗I\right),$$

$$a\;|\;\Lambda_{A}\sim \mathrm{MVN}\left(0,\Lambda_{A}⊗A\right),$$

$$p\;|\;\Lambda_{P}\sim \mathrm{MVN}\left(0,\Lambda_{P}⊗I\right),$$

$$\Lambda_{G}\;|\;v_{g},S_{g}\sim \mathrm{IW}\left(v_{g},v_{g}S_{g}\right),$$

$$\Lambda_{A}\;|\;v_{a},S_{a}\sim \mathrm{IW}\left(v_{a},v_{a}S_{a}\right),$$

$$\Lambda_{P}\;|\;v_{p},S_{p}\sim \mathrm{IW}\left(v_{p},v_{p}S_{p}\right),$$

$$R\;|\;v_{e},S_{e}\sim \mathrm{IW}\left(v_{e},v_{e}S_{e}\right),$$

where *MVN* and *IW* indicate multivariate normal and inverse Wishart distributions, respectively, and $v_{g},S_{g}$, $v_{a},S_{a}$, $v_{p},S_{p}$ and $v_{e},S_{e}$ correspond to the degree of confidence and prior values for contemporary group, additive genetic, permanent environmental, and residual covariances, respectively.

Covariance components were estimated via single‐trait analyses using Bayesian inference. A single chain of 300,000 iterations was generated for each model, with an initial burn‐in period of 100,000 iterations and a thinning interval of 10. Chain convergence was assessed through visual inspection of trace plots across iterations, as well as by the criteria of Geweke (1992) and Raftery and Lewis (1992). Thus, the variance components for the regression coefficients and genetic parameters (heritability and genetic correlation) were estimated from the remaining 20,000 samples.

Heritability for the intercept regression coefficient was estimated as a ratio of additive genetic variance over the sum of contemporary group, additive genetic and permanent environmental variances for the intercept component (Jamrozik et al. 2001).

Once the optimal model for each trait was defined, the contemporary group, additive genetic, permanent environmental, residual and phenotypic variances were estimated for both definitions of FL. The (co)variance components, heritabilities and genetic correlations on the longitudinal scale were estimated using the standard formulae of linear functions of regression coefficients as proposed by Jamrozik and Schaeffer (1997). Heritabilities on the longitudinal scale for FLSTAY and FLNC were estimated as a ratio of additive genetic variance to the sum of contemporary group, additive genetic, permanent environmental and residual variances to the desired age.

The following criteria were used for comparison of the random regression models:

1. Mean squared error (MSE): $\sum_{i=1}^{n}\frac{{\left(y_{\mathrm{oi}}-y_{\mathrm{pi}}\right)}^{2}}{n}$, where $y_{\mathrm{oi}}$ is the *i*‐th observed phenotype, $y_{\mathrm{pi}}$ is the predicted phenotype for observation *i* and *n* is the total number of phenotypic records.
2. Posterior mean of the deviance ($\bar{D}\left(\theta \right)$): the expected value of the deviance over the posterior distribution, defined as $D\left(\theta \right)=-2\ln p\left(y|\theta \right)$, where $p\left(y|\theta \right)$ is the likelihood function of the data evaluated at each MCMC iteration.
3. Deviance information criterion (DIC) proposed by Spiegelhalter et al. (2002): $\mathrm{DIC}=\bar{D}\left(\theta \right)+p_{D}=2\bar{D}\left(\theta \right)+D\left(\bar{\theta }\right)$. The effective number of parameters represents a penalty with the increasing complexity of the model:$p_{D}=\bar{D}\left(\theta \right)-D\left(\bar{\theta }\right)$, where θ is the vector of parameters of the model and $D\left(\bar{\theta }\right)$ is the Bayesian deviance evaluated at the posterior means of the parameters. Lower values for DIC indicate more adequate models.

To evaluate the predictive performance and stability of the estimated breeding values (EBV), a two‐fold cross‐validation scheme was implemented targeting two distinct categories of selection candidates: young bulls and cows. For the young bull scheme, which simulates early sire selection prior to the availability of progeny phenotypes, sires were selected from the complete dataset if they had a minimum of 50 daughters with known phenotypes for each of the first six standard ages from 27 to 87 months, resulting in a validation set of 118 bulls. A partial dataset was then constructed by truncating all records after the year 2007, a specific time point when these 118 target sires had zero daughters recorded in the database. Genetic evaluations were subsequently executed using both this partial dataset, wherein the candidate sires lacked progeny data and relied solely on pedigree information, and the complete dataset, where they were proven. For the cow validation scheme, a subset of cows with complete phenotypic records up to 87 months of age was identified, from which a random sample of 2000 individuals was selected. This sample comprised 1000 high‐performance cows with FLNC values of 5 or 6 at 87 months of age and 1000 low‐performance cows with FLNC values of 1 or 2 at that age. A corresponding partial dataset was generated by masking the phenotypic records of these 2000 cows from 63 months of age onward, and genetic evaluations were conducted across both the partial and complete data structures. For both cross‐validation schemes, genetic evaluations used the posterior means of the (co)variance components for the respective models. Predictive capacity and model bias were quantified by calculating the Pearson correlation coefficient and the linear regression slope of the EBVs at 75 months of age, derived from the complete dataset on those obtained from the partial dataset, where a regression slope close to 1.0 indicates an absence of prediction inflation or deflation. The age of 75 months was chosen for this validation as it closely aligns with the time point utilized for traditional stayability definitions in beef cattle breeding programmes.

Complementary analysis for comparison of the models included the rank correlations between sires, classifying them according to the EBV for the trait at each of the 10 ages.

## 3 Results and Discussion

When analysing the phenotypic means across ages (Table 2), it is important to consider the methodological penalization applied to both traits: for cows that failed to calve for two consecutive years, any subsequent calving records were discarded. Influenced by this constraint, FLSTAY exhibited a peak success rate at 39 months of age (0.72), indicating that most cows had produced their first calf by this point. Following this peak, the mean progressively declined to 0.08 at 135 months. This sharp decrease could reflect the gradual culling of animals, as well as the permanent assignment of failure due to consecutive reproductive gaps. Conversely, the mean for FLNC increased steadily throughout the cow's productive life, as expected for a cumulative trait. However, by 135 months of age, the mean number of accumulated calvings reached only 2.96. Given the biological potential for up to ten calvings within this timeframe, this relatively low average highlights the impact of reproductive failures (which trigger the methodological plateau effect) and a possible high intensity of culling in the evaluated Nellore population.

Within the scope of the evaluated polynomial orders, and conditional on the specific data structure defined by our quality control criteria, the optimal model specification varied according to the trait definition. For FLSTAY, the cubic polynomial model (four coefficients) demonstrated the highest fit, whereas for FLNC, the quadratic model (three coefficients) was selected as superior based on the comparison criteria presented in Table 3. The cubic model applied to FLNC failed to reach a converged solution for the (co)variance components within 300,000 iterations and was consequently excluded from the comparison. This lack of convergence is likely attributable to overparameterization and collinearity among the higher‐order Legendre polynomial coefficients when applied to a relatively simple longitudinal trajectory like that of FLNC (Meyer 1998; Misztal 2006). Based on the premise that model complexity must mirror the dispersion behaviour of the data, Misztal (2006) proposed that the density and placement of knots in linear spline functions should be guided by changes in phenotypic variance. By direct analogy, selecting the polynomial order in orthogonal Legendre functions could adhere to the same structural logic. As observed in the descriptive statistics (Table 2), the phenotypic standard deviation for FLNC exhibits an almost linear increase across the standard ages. Assuming that the additive genetic and permanent environmental variances follow a similarly low‐complexity trend, attempting to fit a cubic function (four random regression coefficients) forces the model to estimate a redundant parameter whose true variance lies at or near the boundary of the parameter space (i.e., zero) (Meyer 1998). In Bayesian inference via Gibbs sampling, this over‐specification induces poor mixing, high autocorrelation, and numerical instability due to near‐singularity in the (co)variance matrices (Sorensen and Gianola 2002), rendering the cubic framework structurally inappropriate for FLNC. Higher‐order models generally provide a better fit to the data due to the increased flexibility of the trend curves, which allows for a more precise adaptation to data variability. However, the increased complexity inherently demands greater computational resources and computing time, while increasing the susceptibility to overfitting and erratic parameter estimates. Therefore, parsimonious models should be favoured when the underlying variance components follow low‐complexity trajectories (Schaeffer 2004).

**TABLE 3:** Mean squared error (MSE), Deviance Information Criterion (DIC), and posterior mean of the deviance ($\bar{D}\left(\theta \right)$) according to the trait definition, functional longevity expressed as stayability (FLSTAY) or functional longevity associated with the number of calvings (FLNC), for the evaluated models.

Model^a^	MSE^b^	DIC^b^	$\bar{D}\left(\theta \right)$ ^b^
FLSTAY			
M2	2.92	255,015	−311,118
M3	0.64	45,897	−55,994
M4	**0**	**0**	**0**
FLNC			
M2	4.41	621,291	−760,273
M3	**0**	**0**	**0**

^a^ M2: Linear model (two coefficients); M3: Quadratic model (three coefficients); M4: Cubic model (four coefficients).

^b^ Values represent the deviation relative to the best‐fitting model (which serves as the baseline of 0).

The predictive performance and stability of the models were assessed through a cross‐validation approach based on estimated breeding values at 75 months of age (Table 4). For the young bull validation scheme, which replicates the real‐world scenario of selecting sires before progeny performance data become available, the FLNC trait demonstrated a slight yet consistent superiority in predictive ability compared to the FLSTAY definition. The highest predictive correlation for young bulls was achieved under the quadratic FLNC model (*r* = 0.61), marginally outperforming the optimal cubic FLSTAY model (*r* = 0.58). Regarding prediction bias, the linear regression slope (*b*) for the quadratic FLNC model was 1.02, indicating a well‐controlled expectation of scale close to the ideal absence of bias (*b* = 1.0), whereas the cubic FLSTAY model exhibited a slightly higher slope (*b* = 1.05). Although these numerical differences between the optimal models of both traits are modest, they systematically favour the FLNC approach.

**TABLE 4:** Cross‐validation metrics (Pearson correlation and linear regression slope) for estimated breeding values at 75 months of age for young bulls and cows according to the trait definition, functional longevity expressed as stayability (FLSTAY) or functional longevity associated with the number of calvings (FLNC), for the evaluated models.

Trait	Evaluation group	Subgroup	Correlation (*r*)			Regression slope (*b*)		
			M2	M3	M4	M2	M3	M4
FLSTAY	Young Bulls (*n* = 118)	Overall	0.541	0.550	0.579	0.930	1.050	1.050
	Cows (*n* = 2000)	Overall	0.950	0.952	0.974	1.230	1.200	1.140
		Bottom 1000	0.971	0.973	0.986	0.993	0.983	1.000
		Top 1000	0.955	0.966	0.967	1.120	1.080	1.060
FLNC	Young Bulls (*n* = 118)	Overall	0.598	0.613	—	0.991	1.020	—
	Cows (*n* = 2000)	Overall	0.976	0.979	—	1.180	1.160	—
		Bottom 1000	0.976	0.974	—	0.971	0.966	—
		Top 1000	0.987	0.992	—	1.060	1.040	—

*Note:* M2: Linear model (two coefficients); M3: Quadratic model (three coefficients); M4: Cubic model (four coefficients). (—): results not available for FLNC/M4 due to lack of convergence.

In the cow validation scheme, where future longitudinal phenotypes were masked from 63 months of age onward, both FL definitions yielded high overall predictive correlations (*r* > 0.95). This high predictability is expected given that the initial calving records for the sample cows remained available in the partial dataset. When partitioning the females into extreme phenotypic subgroups, the differences between the models and traits remained discrete. Within the high‐performance subgroup (Top subgroup, representing cows with five or six calvings by 87 months), the quadratic FLNC model exhibited a slightly higher predictive correlation (*r* = 0.99) combined with a regression slope near unity (*b* = 1.04). Under the binary FLSTAY framework, the optimal cubic model for the same subgroup resulted in a correlation of 0.97 and a regression slope of 1.06. The subgroup analysis revealed a systematic behaviour regarding prediction dispersion across all evaluated trait definitions and model frameworks. For the high‐performance subset (Top subgroup), the regression slopes were consistently greater than unity, demonstrating that early genetic evaluations tend to deflate the EBV variance of superior cows, underestimating their genetic merit due to model shrinkage in the absence of lifetime phenotypes. Conversely, the low‐performance subset (Bottom subgroup) consistently exhibited slopes near or slightly below unity, indicating that early‐life genetic evaluations present controlled dispersion or a minor over‐dispersion (inflation) of genetic differences for inferior cows.

Collectively, the cross‐validation metrics demonstrate that while the numerical differences between the best‐fitting models for FLSTAY and FLNC are relatively discrete, modelling FL as a cumulative trait via random regression provides a more stable framework for early selection decisions. This advantage likely stems from the continuous nature of the cumulative number of calvings, which retains the detailed temporal records of sequential reproductive successes more effectively than collapsing multiple biological outcomes into binary categories, thereby yielding both superior model fit and enhanced predictive stability.

Regarding the statistical framework for FLSTAY, threshold models are conceptually more appropriate for binary data than linear models because they account for the discrete nature of records on an underlying liability scale (Gianola and Foulley 1983). However, several studies in the literature have shown small practical differences between linear and non‐linear approaches when applied to field data (Olsen et al. 1994; Matos et al. 1997). In a simulation study, Jamrozik et al. (2008) demonstrated that linear random regression animal models provided superior predictive ability for the proportion of surviving daughters compared to the proportional hazard model. Using real, large‐scale field data, Jamrozik et al. (2013) formally contrasted linear and threshold RR models for stayability to consecutive calvings and reported a strong superiority of the linear model regarding fitting criteria on the observable scale. From a practical perspective, the linear RR model is highly feasible for routine genetic evaluations of FL, where its computational tractability and numerical stability make it well‐suited for processing large‐scale nationwide databases (Schaeffer 2004) without compromising selection accuracy (Meijering and Gianola 1985).

Investigating a trait similar to FLSTAY, Oliveira et al. (2020) found that a quartic (five coefficients) Legendre polynomial model yielded the best performance for evaluating the trait in Angus cattle. Similarly, when studying longevity based on consecutive calvings without predetermined fixed ages, Silva et al. (2017) and Silva et al. (2021) identified the cubic (four coefficients) model as the most suitable for Zebu and taurine cattle populations. Furthermore, Jamrozik et al. (2013) concluded that the cubic model was the most appropriate for describing stayability to consecutive calvings in Simmental cattle, noting that while the quartic model showed a slight increase in marginal likelihood, it performed poorly across other metrics. Conversely, regarding FLNC, literature evaluating longevity through accumulated calvings using random regression models remains scarce.

All the subsequent results are presented for the best‐fitting models compared, cubic (four coefficients) for FLSTAY and quadratic (three coefficients) for FLNC. For FLSTAY, the estimates for the contemporary group (CG), additive genetic (ADD), permanent environmental (PE), residual (RES) and phenotypic (PHE) variances (Figure 1) generally displayed a similar trajectory. The CG, RES and PHE variances progressively increased from the first to the third or fourth age point and then experienced a gradual decline towards the end of the evaluated age scale. The ADD and PE variances increased until peaking at the sixth age point. This behaviour of the variances is consistent with the binary trait definition. Since failure rates are elevated at both the earliest age (animals that never calve) and the latest age (animals progressively culled due to reproductive failures), variability is inherently restricted during these extremes, peaking only at intermediate ages where the population presents a more balanced distribution of successes and failures. In contrast, for FLNC, all variance components consistently increased as the age advanced. This continuous upward pattern reflects the cumulative nature of the trait definition. Older ages naturally allow for a wider diversity of assigned phenotypes (e.g., cows with 1 up to 10 accumulated calves), continuously expanding the variability over time. This trend also elucidates the superior fit of the quadratic model for FLNC, as it naturally accommodates this continuous variance increment across the age scale.

**FIGURE 1:**
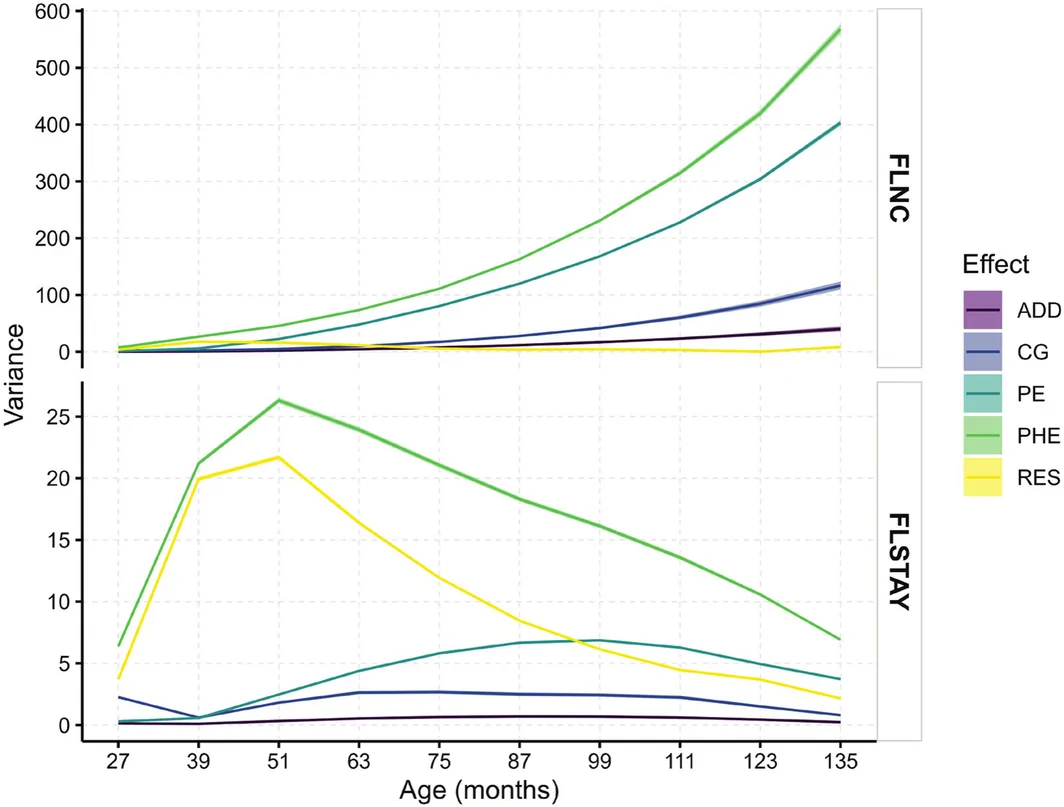
Contemporary group (CG), additive genetic (ADD), permanent environmental (PE), residual (RES) and phenotypic (PHE) variances (95% highest posterior density intervals—shaded) for functional longevity associated with the number of calvings (FLNC) and for functional longevity expressed as stayability (FLSTAY) according to the predetermined age. [Colour figure can be viewed at wileyonlinelibrary.com]

The relative contribution of each variation source to the phenotypic variance was dynamic across the predetermined ages for both FL definitions (Figure 2). Generally, the proportion of residual variance increased from the first to the second calving, but gradually decreased in subsequent ages. Conversely, the relative importance of permanent environmental variance increased as the evaluated age advanced. These trends were more pronounced for FLNC.

**FIGURE 2:**
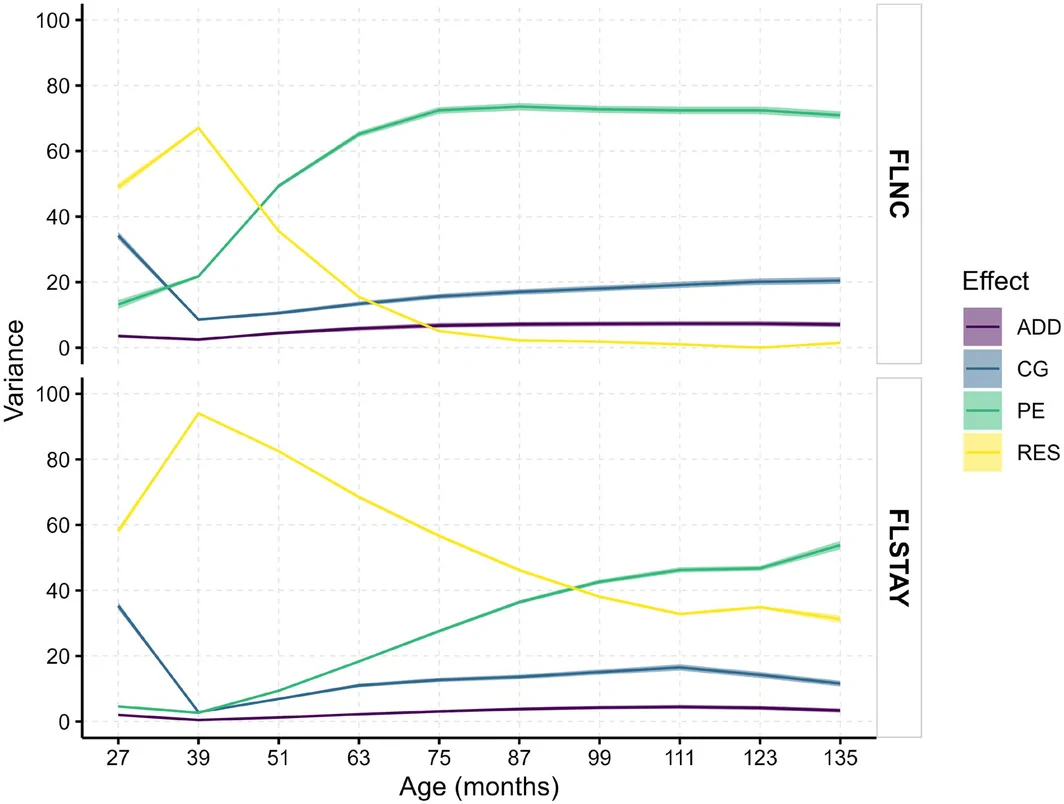
Proportions of contemporary group (CG), additive genetic (ADD), permanent environmental (PE) and residual (RES) variances relative to the phenotypic variance for functional longevity associated with the number of calvings (FLNC) and for functional longevity expressed as stayability (FLSTAY) according to the predetermined age. [Colour figure can be viewed at wileyonlinelibrary.com]

For both trait definitions, the CG contribution dropped from the first to the second predetermined age, stabilizing thereafter until the end of the age scale. Meanwhile, the additive genetic variance remained relatively stable across all ages, showing slightly higher values starting from the fourth predetermined age. Both additive genetic and CG variances had slightly greater relative importance in the FLNC definition.

The interpretation of the estimated genetic and environmental parameters for FLSTAY and FLNC across the longitudinal scale must integrate both mathematical and biological standpoints. Because random regression models fit continuous trajectories over longitudinal records of cumulative calving (FLNC) or stayability (FLSTAY), the variance expressed at any given age represents a transformed expression of the differences in the timing of reproductive failure or herd exit. However, from a livestock production perspective, this timing is not an isolated random event. It reflects the accumulated history of the cow's multi‐trait performance up to that specific stage. In commercial beef cattle operations, culling decisions are dictated by a latent index of economic utility, combining regular calving success with maternal ability (e.g., calf weaning weight), locomotion, and physical soundness (Brzáková et al. 2019; Catrett et al. 2024). Consequently, a phenotype recorded at an advanced age inherently integrates the sequential barriers the animal has successfully overcome throughout its lifespan. This biological cumulative effect explains the expansion of the permanent environmental variance at older ages observed in our study (Figure 1), capturing the long‐term environmental deviations and management decisions that shape the cow's productive trajectory.

When investigating a trait definition similar to FLSTAY in Zebu and taurine cattle breeds, Silva et al. (2018) and Silva et al. (2021) reported variance proportion curves that differed from those observed in the present study. In their studies, the residual variance accounted for lower proportions, ranging from near zero to approximately 25%. Similarly, Jamrozik et al. (2013) reported residual variance proportions between near zero and 22% for Simmental cattle. In contrast, the present study found a much larger proportion of residual variance for FLSTAY, ranging from 31% to 94%. Passafaro et al. (2024) reported that the residual variance accounted for ≈30% to ≈68% of the phenotypic variation in a multibreed beef population. For FLNC, the proportion of residual variance varied from near 0% to 68%.

Another notable discrepancy relates to the proportion of permanent environmental variance. Silva et al. (2018) and Silva et al. (2021) reported proportions ranging from 60% to approximately 90%, while Jamrozik et al. (2013) found values between 40% and 80%. Similarly, Aliloo et al. (2025) observed that permanent environmental variance explained the highest amount of variation across all ages for FL in Australian Angus cattle. In this study, the permanent environmental variance proportions for FLSTAY were considerably smaller, fluctuating between 3% and 47% of the total phenotypic variance.

The heritability of the random regression intercept coefficient ($h_{\alpha 0}^{2}$) was estimated at 0.08 for both FLSTAY and FLNC. In random regression analyses, the intercept coefficient represents the age‐independent average level of the trait across trajectory. Thus, selection for FL using EBVs for this coefficient may result in a favourable change in the average of the FL for the population across all ages studied. Our estimate of $h_{\alpha 0}^{2}$ for FLSTAY was substantially lower than the intercept heritability of 0.24 reported by Jamrozik et al. (2013) for stayability to consecutive calvings in Simmental cattle. On the other hand, our result aligns closely with the findings of Silva et al. (2018), who estimated $h_{\alpha 0}^{2}$ ranging from 0.06 to 0.09 for stayability to consecutive calvings in Zebu cattle.

Heritability estimates for FLNC ranged from 0.025 to 0.074 and were consistently higher than those estimated for FLSTAY, which ranged between 0.004 and 0.045 across age scale (Figure 3). The estimates found in this study indicate that selection for FL can be effective, as there is genetic variability among animals for these traits. The values found for FLSTAY are lower than those reported for stayability to consecutive calvings in Zebu (Silva et al. 2018) and taurine (Jamrozik et al. 2013; Silva et al. 2021) populations. For an FL definition similar to FLSTAY, Oliveira et al. (2020) reported heritability estimates ranging from 0.07 to 0.25, while Aliloo et al. (2025) reported heritability estimates generally below 0.10 in North American and Australian Angus cattle, respectively. Similarly, Passafaro et al. (2024) reported heritability estimates from RRM showing a decreasing trend from 0.16 to 0.04 for a multibreed cattle population. Furthermore, Brzáková et al. (2019) reported heritability estimates below 0.14 for FL defined as the number of calves produced up to four specific ages (e.g., 78, 90, 150 and 160 months) in a multibreed population using single‐trait or multiple‐trait models.

**FIGURE 3:**
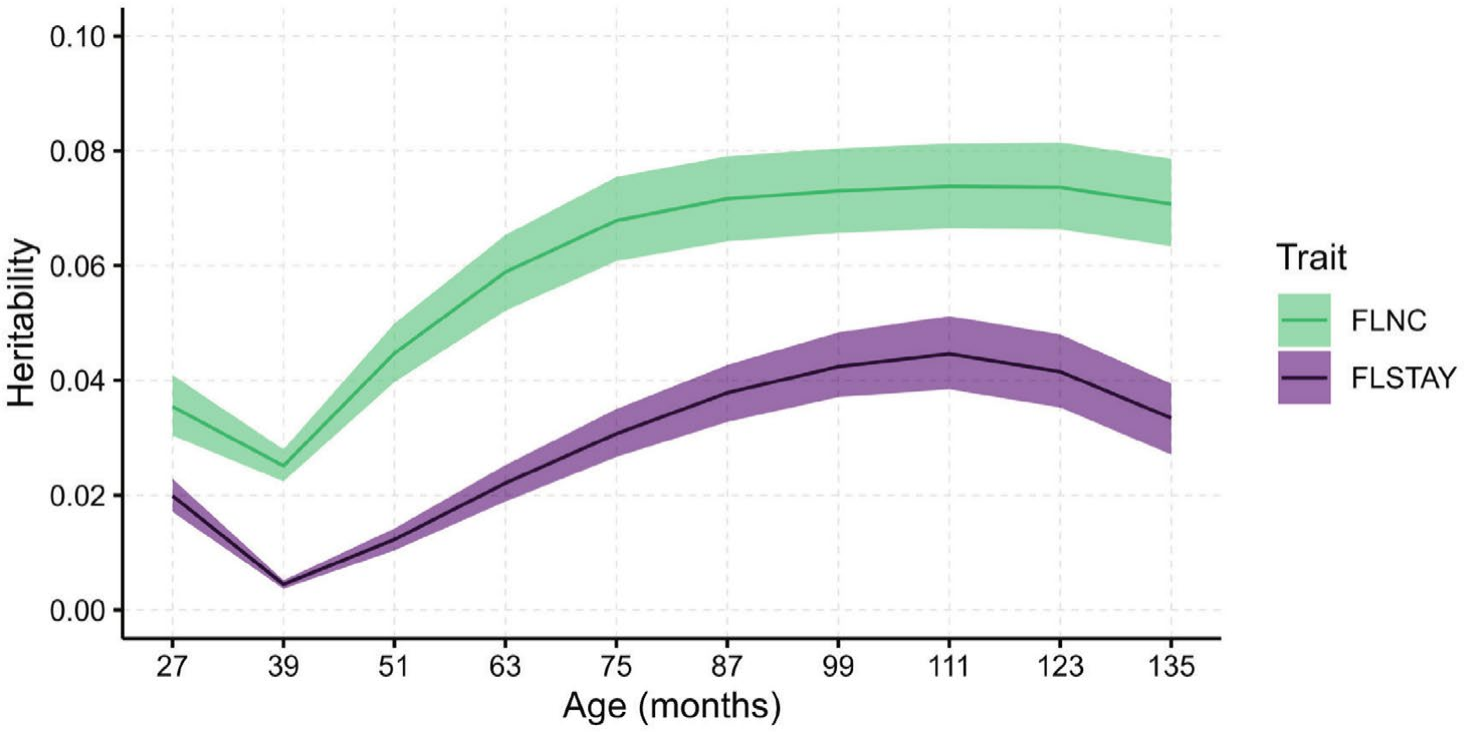
Heritability estimates (95% highest posterior density intervals—shaded) for functional longevity associated with the number of calvings (FLNC) and for functional longevity expressed as stayability (FLSTAY) according to the predetermined age. [Colour figure can be viewed at wileyonlinelibrary.com]

Genetic correlation estimates across different ages were similar for FLNC and FLSTAY (Table 5). For both trait definitions, except for the earliest age class (27 months), strong genetic correlations were observed between FL at different ages, indicating the feasibility of practicing early selection in younger animals (e.g., at 39 months). As expected for longitudinal traits, the genetic correlations were highest between adjacent ages and gradually decreased as the distance between the evaluated ages increased. This typical pattern has been widely reported for stayability to consecutive calvings in Zebu (Silva et al. 2018), and taurine (Jamrozik et al. 2013; Silva et al. 2021) populations.

**TABLE 5:** Mean estimates (and respective 95% highest posterior density intervals in parentheses) for genetic correlations among different ages (months) for functional longevity expressed as stayability (FLSTAY) (above diagonal) and functional longevity associated with the number of calvings (FLNC) (below diagonal).

Age (months)	27	39	51	63	75	87	99	111	123	135
27	—	−0.05 (−0.16 to 0.06)	−0.35 (−0.44 to −0.25)	−0.32 (−0.41 to −0.23)	−0.21 (−0.30 to −0.12)	−0.07 (−0.16 to 0.03)	0.07 (0.02 to 0.18)	0.18 (0.08 to 0.29)	0.21 (0.10 to 0.32)	0.00 (−0.14 to 0.13)
39	0.50 (0.43 to 0.58)	—	0.95 (0.94 to 0.96)	0.94 (0.93 to 0.96)	0.94 (0.92 to 0.96)	0.92 (0.88 to 0.95)	0.87 (0.81 to 0.91)	0.80 (0.73 to 0.87)	0.75 (0.67 to 0.85)	0.73 (0.63 to 0.86)
51	0.22 (0.13 to 0.31)	0.95 (0.94 to 0.96)	—	0.99 (0.99 to 1.00)	0.97 (0.96 to 0.98)	0.91 (0.88 to 0.94)	0.83 (0.78 to 0.88)	0.74 (0.67 to 0.81)	0.69 (0.61 to 0.78)	0.73 (0.63 to 0.86)
63	0.11 (0.02 to 0.20)	0.91 (0.89 to 0.92)	0.99 (0.99 to 0.99)	—	0.99 (0.98 to 0.99)	0.95 (0.93 to 0.96)	0.87 (0.84 to 0.91)	0.80 (0.74 to 0.85)	0.75 (0.68 to 0.82)	0.78 (0.69 to 0.88)
75	0.06 (−0.04 to 0.15)	0.88 (0.85 to 0.90)	0.98 (0.97 to 0.98)	1.00 (1.00 to 1.00)	—	0.98 (0.98 to 0.99)	0.94 (0.92 to 0.95)	0.88 (0.84 to 0.91)	0.84 (0.79 to 0.89)	0.84 (0.77 to 0.92)
87	0.03 (−0.07 to 0.12)	0.85 (0.82 to 0.88)	0.96 (0.95 to 0.97)	0.99 (0.99 to 0.99)	1.00 (1.00 to 1.00)	—	0.98 (0.98 to 0.99)	0.95 (0.93 to 0.96)	0.92 (0.89 to 0.95)	0.89 (0.82 to 0.95)
99	0.01 (−0.09 to 0.10)	0.83 (0.79 to 0.86)	0.95 (0.93 to 0.96)	0.98 (0.97 to 0.98)	0.99 (0.99 to 0.99)	1.00 (1.00 to 1.00)	—	0.99 (0.98 to 0.99)	0.97 (0.96 to 0.98)	0.92 (0.86 to 0.97)
111	0.00 (−0.10 to 0.09)	0.80 (0.76 to 0.84)	0.93 (0.91 to 0.94)	0.96 (0.95 to 0.97)	0.98 (0.98 to 0.98)	0.99 (0.99 to 0.99)	1.00 (1.00 to 1.00)	—	0.99 (0.99 to 1.00)	0.93 (0.88 to 0.97)
123	−0.01 (−0.11 to 0.09)	0.78 (0.73 to 0.82)	0.90 (0.88 to 0.93)	0.94 (0.93 to 0.96)	0.97 (0.96 to 0.97)	0.98 (0.98 to 0.99)	0.99 (0.99 to 0.99)	1.00 (1.00 to 1.00)	—	0.95 (0.92 to 0.98)
135	−0.01 (−0.11 to 0.10)	0.75 (0.70 to 0.80)	0.88 (0.85 to 0.91)	0.92 (0.90 to 0.94)	0.95 (0.94 to 0.96)	0.97 (0.96 to 0.98)	0.98 (0.98 to 0.99)	0.99 (0.99 to 0.99)	1.00 (1.00 to 1.00)	—

The close‐to‐unity genetic correlations estimated across adjacent mature ages for both trait definitions (Table 5) must be interpreted with caution under the properties of stochastic processes. In longitudinal modelling, both FL frameworks used here introduce a history‐dependent structure following reproductive failure or culling events. For FLSTAY, once a cow experiences a culling event or consecutive reproductive failures, subsequent records are fixed to zero, creating an absorbing process. Because the residual covariance structure in these linear random regression models is mathematically constrained to be diagonal, the mixed model equations partition this artificial temporal dependency directly into the random regression effects, potentially forcing the genetic correlations close to unity across later stages. This behaviour likely represents a blend of anticipated biological pleiotropy, since the same sets of genes are expected to govern FL across adjacent mature stages, and a mathematical constraint imposed by data recording structures.

A parallel phenomenon is observed for FLNC, where genetic correlations among mature ages also reach values close to unity between adjacent intervals and 0.95 between 75 and 135 months, for instance (Table 5). Once a cow stops calving, her individual trajectory stabilizes into a deterministic plateau, maintaining repeated identical count records that introduce a form of pseudo‐replication. However, a distinction between the two traits lies in their underlying variance structures. Whereas the absorbing process of FLSTAY collapses all culled individuals into a uniform zero sink, restricting the variance of the observed phenotypes at later ages (see SD in Table 2), the FLNC plateaus vary individually depending on the exact timing of culling. This structural preservation is confirmed by the monotonic increase in the variance of the observed phenotypes for FLNC across the standard ages (see SD in Table 2). Consequently, this allows the orthogonal polynomials to scale variance components on the expanding scale of phenotypic variation in FLNC, rather than forcing them to operate within the contracted parameter space characteristic of FLSTAY.

This divergence in variance dynamics highlights how the random regression framework handles the longitudinal data structure in later stages. Because animals do not experience subsequent reproductive events after consecutive failures or culling, no new information enters the system to re‐rank individuals, which mathematically causes the genetic covariance trajectories to progress in a parallel, locked manner. Crucially, the random regression model effectively isolates the scale of this temporal redundancy through the permanent environmental (PE) effect. As shown by the expansion of the PE variance component (Figure 1), which captures up to 70% to 75% for FLNC or 50% to 55% for FLSTAY of the total phenotypic variation at advanced ages (Figure 2), the PE effect acts as a statistical buffer. It absorbs the longitudinal autocorrelation induced by individual post‐failure plateaus, protecting the additive genetic variance component from scale distortion and keeping its relative proportion stable and controlled across the cow's lifespan for both traits (Figure 2).

The genetic correlation between the second age point (39 months) and the age traditionally used for selection (around 75 months) reached 0.94 for FLSTAY and 0.88 for FLNC (Table 5). While these strong associations imply that genetic selection at 39 months aligns closely with genetic merit at 75 months, enabling early selection strategies, their high magnitude must be interpreted with caution as stated before. Aliloo et al. (2025) reported genetic correlations higher than 0.90 between earlier ages (3 to 5 years) and 6 years of age for FL in Australian Angus. Furthermore, for an FL definition similar to FLSTAY, Oliveira et al. (2020) found an average correlation of 0.40 across different ages when observing animals between 2 and 15 years old; however, when disregarding the extreme age classes and considering only animals between 3 and 12 years of age, the average correlation increased to 0.70. Passafaro et al. (2024) found genetic correlations ranging from 0.43 to 0.99 among FL in different parities. These results confirm that while consecutive ages are highly correlated, early‐ and late‐life longevity are not perfectly identical traits from a genetic standpoint, justifying the use of random regression instead of repeatability models to capture these variations over time.

The rank correlation analysis for the EBVs obtained from the different evaluated models (Table 6) indicates that, although the values are generally high (except at 27 months of age), some re‐ranking of the animals is expected. This outcome reinforces the necessity of selecting an appropriate model for the genetic evaluation of both FLSTAY and FLNC.

**TABLE 6:** Rank correlations between breeding values predicted from different models^a^ for functional longevity expressed as stayability (FLSTAY) and functional longevity associated with the number of calvings (FLNC) according to the predetermined age.

Age (months)	FLSTAY			FLNC
	M2–M3	M3–M4	M2–M4	M2–M3
27	0.55	0.88	0.57	0.82
39	0.99	0.88	0.88	0.90
51	0.96	0.88	0.76	0.89
63	0.94	0.93	0.80	0.88
75	0.93	0.98	0.88	0.88
87	0.94	0.99	0.94	0.87
99	0.95	0.98	0.98	0.86
111	0.96	0.96	0.98	0.85
123	0.98	0.97	0.97	0.83
135	0.95	0.97	0.96	0.82

^a^ M2: Linear model (two coefficients); M3: Quadratic model (three coefficients); M4: Cubic model (four coefficients).

The use of random regression models makes it possible to estimate EBVs for FL at intermediate ages within the evaluated scale. Additionally, incorporating longitudinal phenotypic records provides a flexible framework to predict breeding values across the evaluated lifespan. This approach grants breeding programmes the flexibility to choose which age‐specific EBVs to report to breeders. From a practical standpoint, this framework may facilitate earlier genetic evaluations, potentially contributing to a reduction in the generation interval.

A limitation of the current study lies in the structure of the database, which lacks explicit records detailing the exact dates and primary motivations for each cow's exit from the herd. Consequently, the longitudinal phenotype construction relies on the operational assumption that an animal was culled based on the prolonged absence of subsequent calving records. As demonstrated by Oliveira et al. (2020), genetic and environmental parameters vary across disposal categories, and combining distinct culling motivations without distinction can attenuate selection responses. Although implementing separate routine genetic evaluations for FL under multiple distinct culling causes remains operationally unfeasible for most breeding programmes, systematically recording culling dates and specific disposal categories is necessary. Beyond improving the precision of phenotypic definitions of FL, capturing this information allows breed associations to accurately monitor the proportions of different culling reasons across beef cattle breeds. Therefore, monitoring these specific proportions provides a diagnostic framework for breed associations to identify breed‐specific vulnerabilities, such as structural unsoundness or fertility failures, and design targeted management and selection strategies to effectively handle these production hurdles (Catrett et al. 2024).

## 4 Conclusions

Compared to traditional binary measures of longevity, such as FLSTAY, the cumulative trait of FLNC provides a more robust genetic evaluation for functional longevity in Nellore cattle, demonstrating a superior model fit, improved predictive ability of EBVs, higher heritability, and stronger genetic correlations across the age scale. To properly capture the longitudinal structure of (co)variance components for this cumulative trait, a quadratic (three coefficients) random regression model fitting Legendre polynomials is the recommended approach. Consequently, evaluating FLNC through this methodology enables early selection, which could reduce the generation interval and accelerate genetic progress for functional longevity in beef cattle breeding programmes.

## Funding

This research was supported by Conselho Nacional de Desenvolvimento Científico e Tecnológico (CNPq: 312480/2021‐0) and Coordenação de Aperfeiçoamento de Pessoal de Nível Superior.

## Conflicts of Interest

The authors declare no conflicts of interest.

## Data Availability

The data that support the findings of this study are available from Programa de Melhoramento Embrapa Gado de Corte. Restrictions apply to the availability of these data, which were used under licence for this study. Data are available from the author(s) with the permission of Programa de Melhoramento Embrapa Gado de Corte.
